# SOX2 regulates common and specific stem cell features in the CNS and endoderm derived organs

**DOI:** 10.1371/journal.pgen.1007224

**Published:** 2018-02-12

**Authors:** Daniel W. Hagey, Susanne Klum, Idha Kurtsdotter, Cecile Zaouter, Danijal Topcic, Olov Andersson, Maria Bergsland, Jonas Muhr

**Affiliations:** 1 Ludwig Institute for Cancer Research, Karolinska Institutet, Stockholm, Sweden; 2 Department of Cell and Molecular Biology, Karolinska Institutet, Stockholm, Sweden; Kyoto Sangyo University, JAPAN

## Abstract

Stem cells are defined by their capacities to self-renew and generate progeny of multiple lineages. The transcription factor SOX2 has key roles in the regulation of stem cell characteristics, but whether SOX2 achieves these functions through similar mechanisms in distinct stem cell populations is not known. To address this question, we performed RNA-seq and SOX2 ChIP-seq on embryonic mouse cortex, spinal cord, stomach and lung/esophagus. We demonstrate that, although SOX2 binds a similar motif in the different cell types, its target regions are primarily cell-type-specific and enriched for the distinct binding motifs of appropriately expressed interacting co-factors. Furthermore, cell-type-specific SOX2 binding in endodermal and neural cells is most often found around genes specifically expressed in the corresponding tissue. Consistent with this, we demonstrate that SOX2 target regions can act as cis-regulatory modules capable of directing reporter expression to appropriate tissues in a zebrafish reporter assay. In contrast, SOX2 binding sites found in both endodermal and neural tissues are associated with genes regulating general stem cell features, such as proliferation. Notably, we provide evidence that SOX2 regulates proliferation through conserved mechanisms and target genes in both germ layers examined. Together, these findings demonstrate how SOX2 simultaneously regulates cell-type-specific, as well as core transcriptional programs in neural and endodermal stem cells.

## Author summary

The fine-tuned activities of stem cells are essential for embryonic development and organ maintenance. All types of stem cells share the abilities to self-renew and differentiate into specific cell-types, though the stem cells of different organs have distinct gene expression profiles and competences. Despite these unifying properties, to what extent diverse populations of stem cells utilize similar regulatory mechanisms remains unclear. To address this issue, we map the binding pattern of the key stem cell transcription factor SOX2 in the developing cortex, spinal cord, stomach and lung/esophagus. We find that, even though the core DNA-sequence it targets is similar in all tissues, its binding location is highly divergent and reflects the gene expression profile of each cell type. Moreover, DNA-regions targeted by SOX2 are enriched for binding motifs of distinct co-factors in each tissue, which we demonstrate can physically and functionally interact with SOX2. Consistent with these findings, DNA-regions bound specifically in cells of one germ layer are capable of driving fluorescent reporter gene expression in corresponding zebrafish tissues. Finally, we demonstrate that proliferation genes are bound by SOX2 in all examined tissues, and that cell division is controlled via a common mechanism in neural and endodermal cells. These findings provide a striking example of how a single transcription factor regulates both core and specific stem cell processes in multiple cellular contexts.

## Introduction

Stem cells are essential for the formation of organs during development, as well as for their homeostasis and maintenance throughout life. Stem cells are defined by their capacity for self-renewal and their ability to generate progeny that differentiate into one or more definitive cell types. However, whether the common characteristics of stem cells are regulated through conserved transcriptional mechanisms in different stem cell populations is not well understood.

The transcription factor SOX2 is expressed in an array of stem cell subtypes, from pluripotent stem cells in the early pre-implantation embryo to adult organ specific stem cells [1]. Gain- and loss-of-function studies have demonstrated that SOX2 has key functions in regulating the fundamental processes of stem cells, including their maintenance, proliferation and cell fate decisions [2–6]. Understanding how SOX2 achieves these distinct functions has been assisted by genome-wide binding analyses, which have revealed thousands of target genes bound by SOX2 in different populations of stem cells [4,7–11]. These experiments have demonstrated that the target selection of SOX2 diverges extensively to specify appropriate gene expression in different stem cell populations, even within the same organ [7].

The binding pattern of SOX2 is influenced by several regulatory mechanisms. While the capacity of SOX2 to target its binding motifs can be influenced by the local status of chromatin compaction [7,12], its binding stability to DNA and target gene selection are also specified by the presence or absence of collaborative partner transcription factors [7,13]. Furthermore, the regulatory capacity of SOX2, and presumably its binding pattern, has been demonstrated to be affected by its level of expression [4,14]. For instance, in the developing cortex high levels of SOX2 maintain uncommitted neural progenitor cells (NPCs) in a slowly proliferating stem cell state by repressing the cell cycle regulator Cyclin D1. Upon commitment to differentiation, the levels of SOX2 decrease, which releases this repression and thus promotes cell cycle re-entry and NPC proliferation [4]. Additionally, SOX2 is also expressed in the endoderm of the anterior foregut that will form part of the respiratory and digestive systems [15]. Interestingly, here SOX2 has been shown to act in a dose-dependent manner to regulate the morphogenesis of the trachea and esophagus [16] [17], and to reduce the capacity of K-RAS to induce bronchiolar tumor formation [18]. However, despite these findings it is still not known how the binding pattern of SOX2 reflects the expression of general and cell-type-specific stem cell features.

To examine how specific and shared gene expression patterns are regulated in different stem cell populations, we have analyzed SOX2 binding in stem cells of the CNS and two endoderm derived organs; the developing lung/esophagus and stomach. We demonstrate that SOX2 targeted DNA-regions are mainly cell-type-specific and enriched for the unique binding motifs of specific co-factors. Moreover, we provide evidence that SOX2 targeted regions can function as cis-regulatory modules (CRMs) that can drive reporter gene expression in corresponding regions of transgenic zebrafish embryos. While cell-type-specific SOX2 binding is enriched around genes with corresponding cell-type-specific expression and function, sites that are bound by SOX2 in both neural and endodermal cells are more often associated with genes that regulate general stem cell features, such as stem cell proliferation. Thus, we use functional experiments to show that the mechanisms by which SOX2 controls the proliferation rate of stem cells in the cortex are conserved in stem cells of the embryonic stomach and spinal cord.

## Results

### SOX2 displays distinct binding profiles in neural and endodermal stem cells

**Fig 1:**
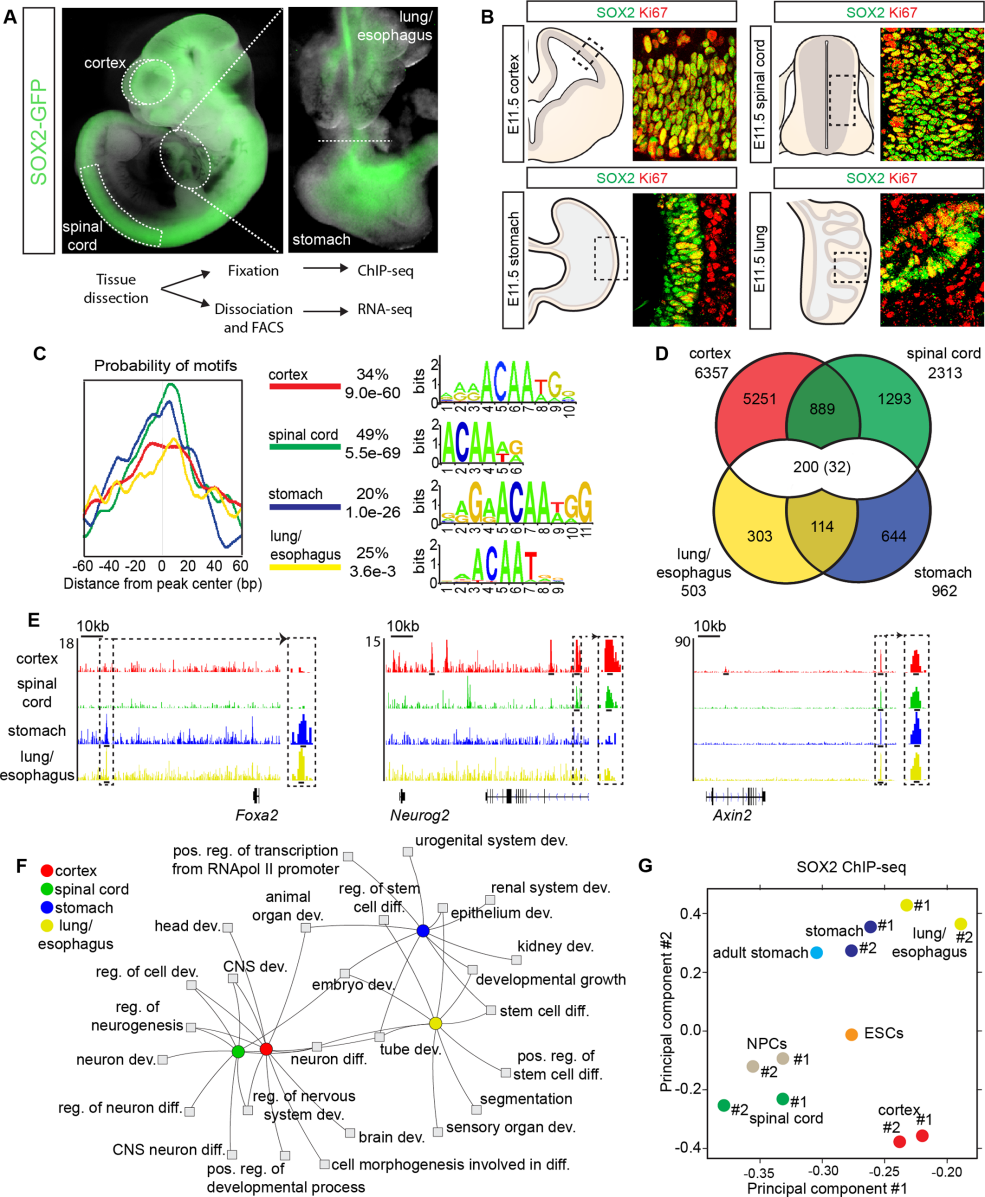
Cell-type-specific binding of SOX2 in CNS and endoderm progenitors. (**A**) E11.5 *Sox2-Gfp* knock-in embryo shows expression in the cortex and spinal cord, while inset highlights expression in dissected stomach and lung/esophagus. (**B**) Overlapping expression of SOX2 and Ki67 in cycling progenitors of the E11.5 cortex, spinal cord, stomach and lung. Grey areas in diagrams represent SOX2^+^ stem cells, while tan surroundings in stomach and lung represent non-endodermal derived mesenchymal cells. (**C**) Central enrichment of SOX2 binding motifs in cortex, spinal cord, stomach and lung/esophagus SOX2 ChIP-seq peaks. The percentage of peaks that the given motifs are found centrally enriched, and their p-values are listed. (**D**) Venn diagram showing overlap between sites bound in the different SOX2 ChIP-seq experiments. 32 peaks overlapped in all four tissues. (**E**) Representative tracks showing germ-layer-specific and common SOX2 ChIP-seq reads aligned around *Foxa2*, *Neurog2* and *Axin2* gene loci, with magnification of selected peak sites. Tracks from cortex are in red, spinal cord in green, stomach in blue and lung/esophagus in yellow, with read scale maximum labelled on y-axis. Inset black lines are centred on peaks. (**F**) Toppcluster Fruchterman-Reingold Network of the top ten GO terms enriched for genes bound by SOX2 in the cortex, spinal cord, stomach and lung/esophagus. (**G**) Diffbind PCA of SOX2 ChIP-seq duplicates in cortex, spinal cord, stomach and lung/esophagus, as well as published SOX2 ChIP-seqs in ESCs, ESC derived NPCs and adult stomach.

Before comparing the roles of SOX2 in different stem cell populations, we confirmed its expression at different axial levels of the central nervous system (CNS) and the foregut, in E11.5 *Sox2-Gfp* knock-in mice (Fig 1A). In the spinal cord, cortex, lung and stomach, the vast majority of neural and endodermal cells expressing the proliferation marker Ki67 were SOX2^+^ (Fig 1B and S1A Fig) [1,2,19,20]. In order to characterize the extent to which SOX2 targets overlap in neuroectodermal and endodermal precursor cells, we performed SOX2 ChIP-seq experiments on dissected E11.5 mouse lung/esophagus and stomach (Fig 1A), and compared this data to publically available *in vivo* SOX2 ChIP-seq experiments from E11.5 mouse cortex and spinal cord cells (S1 Table) [4,7]. For reference, we also performed RNA-seq to examine the gene expression profiles of SOX2-GFP^+^ cells isolated from E11.5 mouse cortex, spinal cord, stomach and lung/esophagus (Fig 1A and S1B Fig).

The ChIP-seq experiments, performed in duplicate, revealed 503 high confidence, consensus SOX2-bound regions (peaks) in lung/esophagus, 962 in stomach, 6357 in cortex and 2313 in spinal cord (Fig 1D and S2 Table), numbers which may reflect the relative abundance of SOX2^+^ cells in each organ, as opposed to their complete set of binding sites. Despite the sequence similarity of the SOX2 target motifs centrally enriched in the different peak sets, region overlap revealed that SOX2 binding was primarily cell-type-specific (Fig 1C and 1D). Although the stringency of our peak calling likely increased the apparent cell-type-specificity, as read density clustering analysis suggested greater overlap between the different SOX2 ChIP-seq experiments (S1C Fig), only a minority of SOX2 ChIP-seq reads within peak regions called as cell-type-specific arose from inappropriate tissues (S1D Fig). In contrast, the regions of SOX2 binding that did overlap were most found within cells of the same germ layer, and only 232 peaks were present in both germ layers, with 32 peaks in all four cell types examined (Fig 1D and 1E and S1C and S2A Figs and S3 Table). Moreover, the relationships between SOX2 binding in the different tissues were also confirmed functionally, as a network map of the top gene ontology (GO) terms [21] enriched for genes bound by SOX2 showed a higher degree of interrelationship within germ layers than between them (Fig 1F). Finally, we confirmed that the SOX2 binding pattern revealed was not due to our peak-calling approach, as peaks re-called using MACS14 generated very similar central motif enrichment, peak numbers and overlaps between the different binding profiles (S2B–S2D Figs and S4 Table)

Consistent with these findings, principal component analysis (PCA) exposed a close relationship between the binding patterns of SOX2 in embryonic endodermal cells and that previously described in the adult stomach [19] (Fig 1G). In contrast, SOX2 binding in the spinal cord and cortex were more closely related to that in embryonic stem cell (ESC) derived NPCs (Fig 1G). Interestingly, the binding pattern of SOX2 in pluripotent ESCs [9] overlapped to a similar extent in all embryonic tissues and separated in between neural and endodermal cells in the PCA (Fig 1G and S3A Fig). Furthermore, by comparing SOX2 binding in ESCs with SOX2 binding in endodermal and neural tissues, we found that SOX2 peaks in ESCs overlapped mostly with peaks common to both germ layers (S3B Fig). Moreover, regions bound by SOX2 in ESCs that were preferentially targeted in only one germ layer were enriched around genes with cell-type-specific functions (S3C and S3D Fig).

### SOX2 binds tissue specific and common stem cell regulatory genes

**Fig 2:**
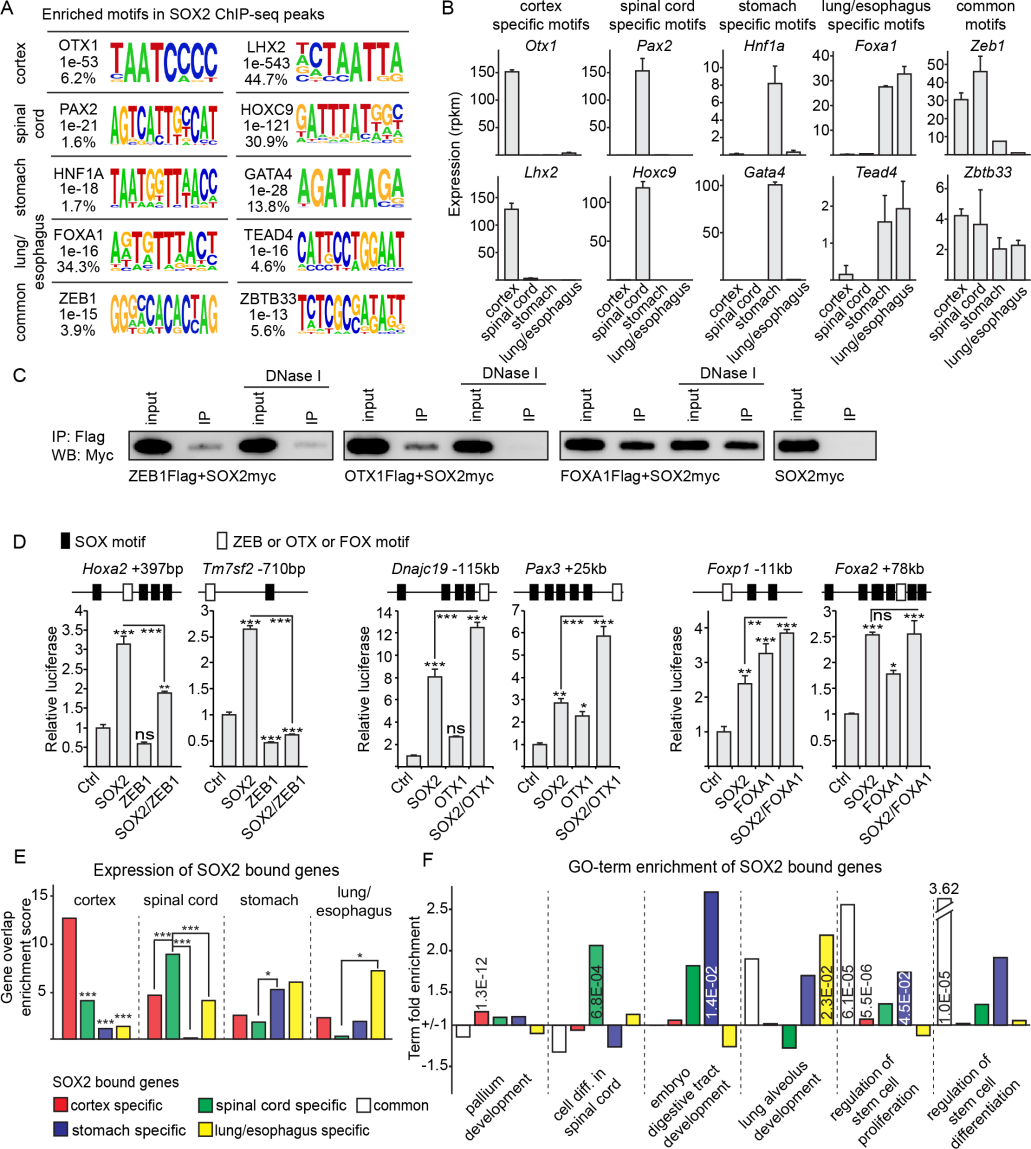
SOX2 utilizes distinct partner factors to drive cell-type-specific gene expression. (**A**) HOMER *de novo* transcription factor binding motifs enriched in specific and common SOX2 ChIP-seq peak sets. Mammalian transcription factors with consensus sites matching the motif, p-values for motif enrichment and the percentage of peaks the motifs are found in are inset next to each motif. (**B**) RNA-seq RPKMs in cortex, spinal cord, stomach and lung/esophagus of transcription factors matching the motifs enriched in HOMER analysis from Fig 2A. (**C**) Co-immunoprecipitation of Flag-tagged transcription factors, identified in Fig 2A as enriched in cortex specific (OTX1), lung/esophagus specific (FOXA1) or common (ZEB1) SOX2 peaks, and Myc-tagged full-length SOX2 with and without DNase I treatment. (**D**) Luciferase assays in P19 cells of common (*Hoxa2* +296bp and *Tm7sf2* -918bp), CNS specific (*Dnajc19* -23kb and *Pax3* +29kb) or endoderm specific (*Foxp1* +98kb and *Foxa2* +98kb) SOX2 bound regions, with depictions of motif arrangements within each enhancer above each assay. Each DNA-region was tested for the effect of co-transfected SOX2 and the specific transcription factor with enriched binding motifs as identified in Fig 2A (OTX1 for neural, FOXA1 for endodermal and ZEB1 for common). P-values are calculated with two-sided, unpaired t-tests. (**E**) Enrichment scores for overlap between genes specifically expressed by Deseq2 analysis between organ RNA-seqs, and genes specifically bound by SOX2 within 500kb in cortex, spinal cord, stomach or lung/esophagus. P-values are calculated by a Yates Chi-squared test. (**F**) Fold enrichment and p-value scores from Panther of selected GO terms for genes within 500kb of each specific and common ChIP-seq peak set. * = p<0.05, ** = p<0.01, *** = p<0.001.

The binding stability and specificity of SOX2 is dependent on its interactions with heterodimerizing partner factors [13]. For instance, the binding pattern of SOX2 has previously been shown to be regulated by its interaction with LHX2 in the cortex and with HOX-proteins, and their associated co-factors, in the spinal cord [7]. Thus, one possibility is that the specific binding profiles of SOX2 in endodermal and neural tissues could be explained by collaboration with distinct partner factors. To address this idea, we assayed DNA-regions specifically or commonly bound by SOX2 in the neural and endodermal cell types for the enrichment of unique transcription factor binding motifs. Apart from previously identified target motifs [7], DNA-regions bound by SOX2 specifically in the cortex were enriched for OTX1 motifs, while those in the spinal cord were enriched for PAX2 motifs (Fig 2A and 2B and S4A Fig). In the endoderm, DNA-regions targeted specifically by SOX2 in the stomach were enriched for motifs targeted by the relevantly expressed transcription factors GATA4 and HNF1A (Fig 2A and 2B and S4A Fig), while those targeted in the lung/esophagus included the relevantly expressed transcription factors FOXA1 and TEAD4 (Fig 2A and 2B and S4A Fig). Finally, regions commonly bound by SOX2 in neural and endodermal cells were instead enriched for ZEB1 and ZBTB33 binding motifs (Fig 2A and 2B and S4A Fig). However, it is important to note that not all enriched motifs may be bound by the transcription factors suggested. For example, HOXD10, which matched a target motif enriched in lung/esophagus specific peaks, is not expressed in cells of these tissues and thus alternative HOX proteins may instead bind these motifs.

The selective enrichment of these binding motifs in the various SOX2 peak-sets raised the question if their corresponding transcription factors could functionally interact with SOX2. To address this question, we focused on OTX1, FOXA1 and ZEB1, which represent transcription factors targeting motifs with distinct spacing to SOX2 motifs (S4B Fig), and whose expression is enriched in neural cells (OTX1), endodermal cells (FOXA1) or more generally within neural and endodermal cells (ZEB1) (Fig 2B). Indeed, co-immunoprecipitation experiments revealed that SOX2 could interact with both FOXA1 and ZEB1 through its C-terminal region, and with OTX1 through its DNA-binding HMG-domain and group B homology domain (HMG+B-domains) (S4C Fig). Moreover, to examine if the detected interactions between these proteins were dependent on linking DNA, we re-examined the interaction between the full-length proteins after DNase I treatment. Interestingly, while we were able to detect interactions between SOX2 and FOXA1, and SOX2 and ZEB1 under these conditions, DNase I treatment completely abrogated SOX2’s interaction with OTX1 (Fig 2C). Although these analyses are indicative of a direct physical interaction between SOX2 and FOXA1 and between SOX2 and ZEB1, it is important to point out that the experiments were based on misexpressed proteins *in vitro*, which raises the possibility that additional undefined factors may facilitate the detected interaction between these transcription factors.

To examine whether these factors could interact at the functional level, CRMs specifically or commonly bound by SOX2 in neural and endodermal tissues, were isolated and inserted into luciferase (luc) reporter vectors. Luc-reporters containing CRMs bound by SOX2 specifically in the CNS were activated in an additive fashion by SOX2 and OTX1 misexpression in mouse embryonic carcinoma P19 cells (Fig 2D). Similarly, CRMs bound by SOX2 in the stomach could be weakly activated both by SOX2 and FOXA1, though these transactivation studies did not reveal any additive effect (Fig 2D). In contrast, while CRMs commonly bound by SOX2 in neural and endodermal tissues were activated by SOX2, this activation was efficiently repressed by co-expressed ZEB1 (Fig 2D). Together, these experiments demonstrate distinct physical and functional interactions between SOX2 and transcription factors targeting motifs enriched in CRMs specifically or commonly bound by SOX2 in neural and endodermal tissues.

In order to study the effects of distinct SOX2 binding profiles, we next examined how the binding pattern of SOX2 correlated with the gene expression profiles of neural and endodermal tissues. The RNA-seq replicates of SOX2-GFP^+^ cells isolated from the cortex, spinal cord, stomach and lung/esophagus of *Sox2-Gfp* mice showed high internal concordance (S4D Fig) and a Deseq2-based comparison of the genes expressed in each tissue revealed a greater overlap in gene expression between cells of the cortex and the spinal cord, or between those of the lung/esophagus and stomach, than between cells of different germ layers (S4E Fig). Moreover, genes found to be specifically expressed showed high scores for appropriate GO terms, such as “*cerebral cortex development*”, “*cell differentiation in spinal cord*”, “*embryo digestive tract morphogenesis*” and “*lung alveolus development*” (S4F Fig and S5 Table). However, even though we isolated SOX2-GFP^+^ cells from the different organs, we cannot exclude the possibility of contaminating non-neural or non-endodermal cells. For instance within SOX2-GFP^+^ lung/esophagus cells isolated by FACS, we could still detect low levels of *Tbx5* expression, which was previously reported to be confined to the lung mesenchyme [22]. Nevertheless, consistent with the cell-type-specific binding pattern of SOX2 in neural and endodermal cells, correlating genes targeted specifically by SOX2 (within 500 kb of closest transcriptional start site) in the cortex (2944 genes), spinal cord (978 genes), stomach (564 genes) or lung/esophagus (252 genes), with the genes differentially expressed in these tissues (S4E Fig and S5 Table), showed that cell-type-specific SOX2 binding was significantly enriched around genes specifically expressed in the corresponding tissue (Fig 2E and S4G Fig). In line with these findings, genes bound by SOX2 in a cell-type-specific fashion were enriched for appropriate cell-type-specific GO terms, such as “*Pallium development*” for cortex bound genes, “*Cell differentiation in spinal cord*” for spinal cord bound genes, “*Embryo digestive tract development*” for stomach bound genes and “*Lung alveolus development*” for lung/esophagus bound genes (Fig 2F). This contrasted with genes commonly bound by SOX2 in neural and endodermal cell types, which were more highly enriched for more generic stem cell GO terms, such as “*Regulation of stem cell proliferation*” and “*Regulation of stem cell differentiation*” (Fig 2F). Hence, compared to genes bound by SOX2 in a cell-type-specific manner, genes commonly bound by SOX2 in neural and endodermal cells were enriched for genes involved in regulating stem cell proliferation and differentiation.

### SOX2-bound CRMs can direct gene expression in a tissue specific fashion

**Fig 3:**
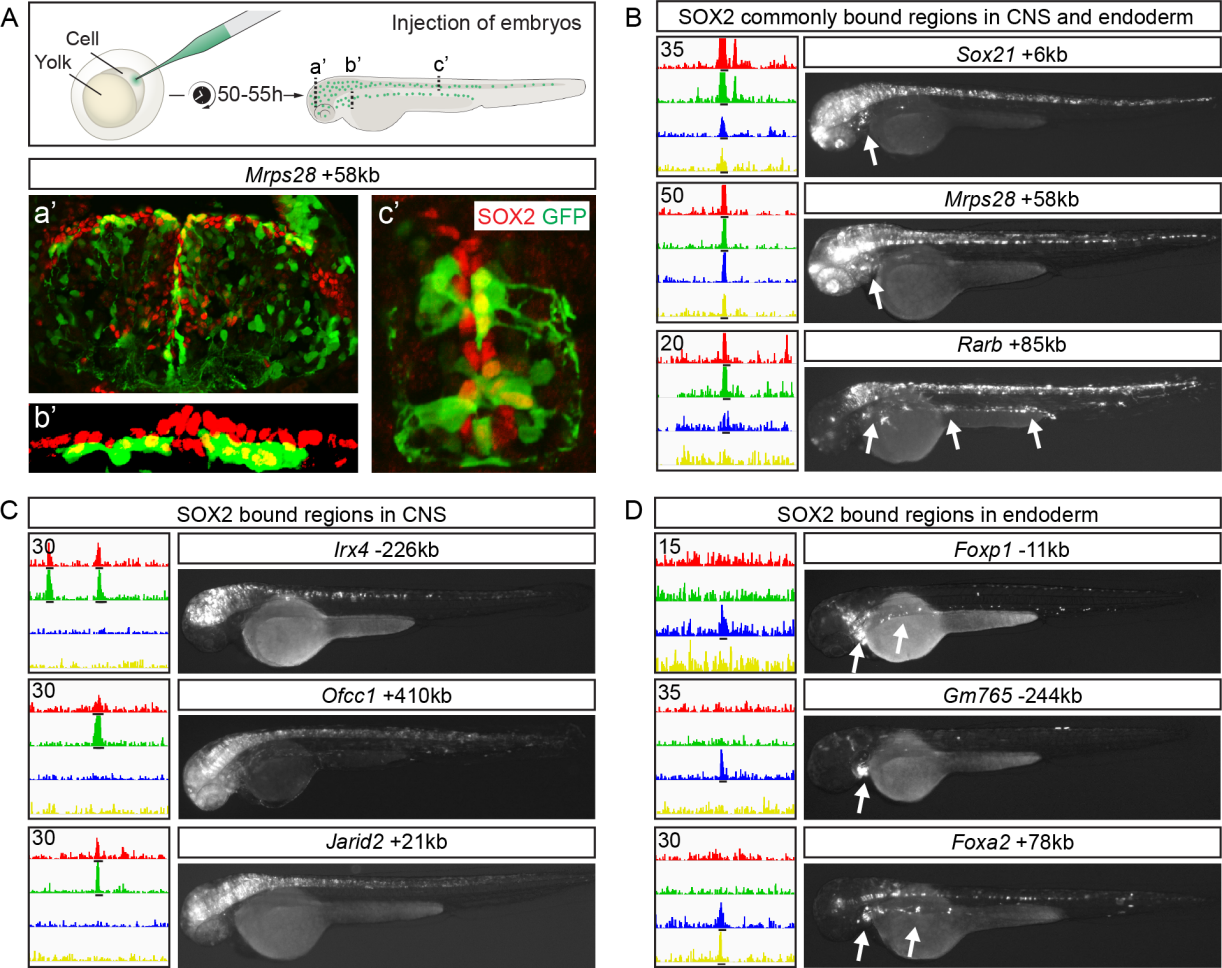
SOX2 bound CRMs possess cell-type-specific enhancer activity. (**A**) Schematic of the zebrafish reporter system used to analyze enhancer expression. Sections of 50 hour post-fertilization zebrafish embryos from three different anterior-posterior levels (a’: midbrain, b’: pharyngesophageal endoderm, c’: spinal cord) following injection of the *Mrps28*+58kb enhancer. Expression of SOX is shown in red and GFP in green. (**B-D**) Three examples each of SOX2 ChIP-seq tracks with read scale maximum values inset top left (cortex in red, spinal cord in green, stomach in blue and lung/esophagus in yellow) and reporter expression from enhancers commonly (B) or specifically bound by SOX2 in CNS (C) and endoderm (D). Arrows point to GFP expression in endoderm. Black lines under tracks indicate called SOX2 peaks.

The finding that the binding profile of SOX2 in neural and endodermal cells reflected the expression patterns of associated genes raised the possibility that SOX2-bound DNA-regions function as CRMs with cell-type-specific activities. To address this possibility, a selection of DNA-regions bound by SOX2 in neural cells, in endodermal cells or in both tissues (for selection of SOX2-bound DNA-regions see Methods), were inserted upstream of a minimal promoter in Tol2-e1b-GFP reporter vectors that were subsequently injected into zebrafish eggs for random integration into the genome (Fig 3A). Of the regions bound by SOX2 commonly in the cortex, spinal cord, stomach and lung/esophagus, 11 out of 12 activated GFP expression in both zebrafish neural and endodermal cells [23] (Fig 3A and 3B and S5A Fig). Furthermore, of the reporters containing DNA-regions bound by SOX2 in mouse neural cells, 5 out of 7 activated GFP expression predominately in the zebrafish CNS (Fig 3C and S5B Fig). Similarly, 4 out of 7 DNA-regions bound by SOX2 in the endoderm activated GFP expression predominately in the zebrafish endoderm (Fig 3D and S5C Fig). Together, these findings demonstrate that genomic regions bound by SOX2 in mouse neural and endodermal cells can function as CRMs that activate gene expression in the corresponding tissue of the developing zebrafish embryo.

### The level of SOX2 expression is a determinant of stem cell proliferation

**Fig 4:**
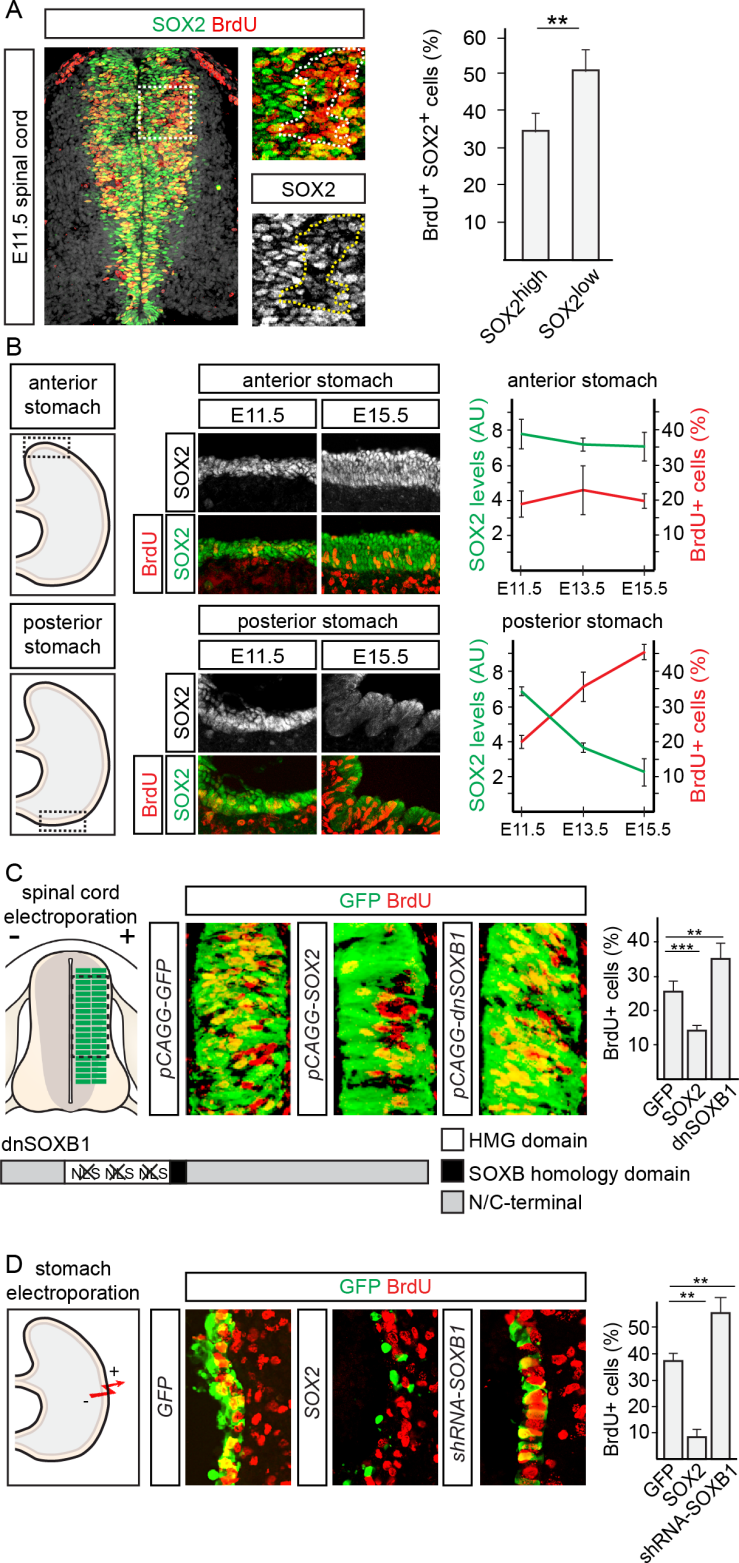
SOX2 represses proliferation in the developing spinal cord and stomach. (**A**) Percentage of cells expressing high or low levels of SOX2 labelled by a one hour pulse of BrdU in the E11.5 mouse spinal cord. Dotted lines in insets surround area of greatest BrdU incorporation. (**B**) Average background normalized SOX2 expression level and percentage of cells labelled by a one hour pulse of BrdU in the E11.5, E13.5 and E15.5 anterior and posterior stomach. (**C**) Percentage of electroporated cells in the chick spinal cord labelled by a 30 minute pulse of BrdU following misexpression of GFP, SOX2 or dnSOXB1. (**D**) Percentage of electroporated cells in E13.5 stomach explants labelled by a 30 minute pulse of BrdU following overexpression of GFP, SOX2 or dnSOXB1. All error bars represent standard deviations between experiments and p-values are calculated with two sided, unpaired t-tests (* = p<0.05, ** = p<0.01, *** = p<0.001).

SOX2 has previously been shown to act in a dose-dependent manner to control the rate of neural precursor cell proliferation in the developing mouse cortex, through the suppression of Cyclin D1 expression [4]. In this respect, it is interesting to note that genes commonly bound by SOX2 in neural and endodermal cells were enriched for GO terms such as stem cell proliferation (Fig 2F) and that the SOX2 responsive *Ccnd1* promoter is targeted by SOX2 in both endodermal and neural cells (S6A Fig). However, whether SOX2 can act in a dose-dependent fashion to regulate stem cell proliferation outside the developing cortex is not known. To address this issue, we began by correlating SOX2 expression levels and cell proliferation in the E11.5 mouse spinal cord (Fig 4A). When divided into two groups based on SOX2 expression levels, the fraction of cells expressing low levels of SOX2 that were labelled by a one hour pulse of BrdU was 1.6-times greater than that of the cells expressing high levels of SOX2 (Fig 4A). In comparison, SOX2 was expressed throughout the stomach endoderm at early developmental stages, but was gradually downregulated posteriorly [17]. Correlating SOX2 expression levels and endodermal cell proliferation in the anterior stomach between stages E11.5 and E15.5 revealed that SOX2 expression and the fraction of BrdU^+^ cells did not change between these stages (Fig 4B). In contrast, the decrease in SOX2 expression in the posterior stomach at E15.5 was paralleled by a significant increase in the fraction of BrdU^+^ endodermal precursor cells and the formation of a striated, undulating epithelium (Fig 4B). Hence, as in the mouse cortex, precursor cells in the spinal cord and stomach that express low levels of SOX2 are generally more proliferative than cells expressing high levels of SOX2.

To examine if the level of SOX2 expression was also instructive in regulating the proliferation rate of these two different precursor cell types, we altered its expression level *in vivo*, using tissue electroporation. This was achieved by either overexpressing SOX2 or by decreasing its activity through the misexpression of a dominant negative SOXB1 protein (dnSoxB1) [4,24] in the chick spinal cord or through the misexpression of shRNAs targeting *SoxB1* mRNAs [4] in the mouse stomach endoderm. In comparison with GFP electroporated cells, overexpression of SOX2 for 20 hours decreased the percentage of cells labelled by a pulse of BrdU, from 25% to 13% in the spinal cord (Fig 4C) and from 35% to 7% in the stomach (Fig 4D). In accordance with these results, decreasing SOX2 activity increased the fraction of BrdU^+^ transfected cells by approximately 50% in the spinal cord and the stomach endoderm (Fig 4C and 4D). Hence, while high levels of SOX2 reduced the number of proliferating cells, decreased expression/activity of SOX2 had the opposite effect and increased the fraction of BrdU incorporating cells.

Interestingly, high levels of Sox2 have previously been shown to reduce cortical proliferation by facilitating TCF/LEF, the Wnt-signaling mediating transcription factors, repression of pro-proliferative genes in a GRO/TLE co-repressor dependent manner [4]. Consistently, high levels of SOX2 could not decrease proliferation in either the spinal cord or in the stomach in the presence of a GRO/TLE binding deficient variant of LEF1 (LEF^GBM^) [25] (S6B and S6C Fig). Conversely, decreased SOX2 activity did not increase proliferation in the presence of a dominant negative version of Tcf7L2 (dnTcf7L2), which cannot recruit the transcriptional activator protein β-catenin [26] (S6B and S6C Fig). Together, these results suggest that SOX2 regulates proliferation via a similar mechanism in the spinal cord and stomach to that previously described in the cortex [4].

## Discussion

SOX2 has key regulatory roles in many different stem cell populations at both embryonic and adult stages. However, whether SOX2 utilizes similar mechanisms to control common cellular processes in different stem cell populations is not understood. To address this issue, we have analyzed SOX2 binding and gene expression, at a genome wide scale, in embryonic mouse cortex, spinal cord, stomach and lung/esophagus. Together, these data reveal a core SOX2 regulated gene network centered on the regulation of stem cell proliferation and differentiation.

An important feature of stem cells is their capacity to be maintained in a slowly proliferative state [27–29]. In this respect, it is interesting that so many of the genes commonly bound by SOX2 in neural and endodermal cells are cell cycle regulators (S3 Table), and that these gave a high enrichment score for the GO-term “*regulation of stem cell proliferation*” (Fig 2F). Moreover, conducting genome-wide studies together with epistatic experiments in the developing mouse cortex, we have previously shown that SOX2 maintains cortical stem cells in a slowly self-renewing state via the repression of Cyclin D1 [4]. In this study, we have extended this finding by showing that the promoter region of *Ccnd1* is bound by SOX2 in all four tissues analyzed here, and that SOX2 decreases proliferation of both neural cells of the spinal cord and endodermal cells of the stomach. As in the cortex, SOX2 appears to achieve this function by counteracting the activity of Wnt/β-catenin signaling [4]. In line with this finding, previous loss-of-function studies in mice have demonstrated that SOX2 suppresses gastric tumorigenesis by dampening hyper-activated Wnt/β-catenin signaling in cells harboring mutations in the tumor suppressor APC [19]. As Wnt/β-catenin signaling is a driver of proliferation in several stem cell niches [26,30,31], it is tempting to speculate that suppressing its activity is a core mechanism by which SOX2 maintains cells in a slowly proliferative stem cell state.

Although the binding pattern of SOX2 is more similar in cells of the same germ layer, most chromatin regions are targeted by SOX2 in a cell-type-specific manner. Due to the inability of SOX2 to stably bind DNA in the absence of partner factor proteins [32], one possible explanation for the region specific binding pattern of SOX2 is the restricted expression of necessary partner factors [13]. Consistent with this idea, we found an enrichment of known transcription factor binding motifs within cell-type specific SOX2-bound regions (Fig 2A), and our RNA-seq analysis further demonstrated an appropriate expression pattern of the factors targeting these motifs in endodermal and neural cells. Moreover, not only have LHX2 and HOXC9 motifs been shown to be necessary for the *in vivo* activity of cortical and spinal cord SOX2-bound CRMs, respectively [7], but SOX2 has previously been shown to interact with the majority of factors targeting the enriched motifs, including members of the POU, PAX, LHX, HOX, MEIS, FOX, GATA, ZBTB, ZEB and PBX families [7,32–34]. Interestingly, our functional analysis shows that these factors, apart from interacting physically, can have diverse effects on SOX2 activity. Thus, it is likely that the transcription factor binding motifs we identify in cell-type-specific SOX2 peaks are important for directing the proper binding pattern and region specific activities of SOX2 in endodermal and neural cells. Moreover, using a transgenic zebrafish system we demonstrate that the activity of SOX2-bound CRMs reflects the tissue specific binding pattern of SOX2 with great accuracy. One important mechanism to control their region specific activities is the discrete expression of interacting transcriptional regulators, such as SOX2 and its partner factors. Together our findings highlight the importance of regulating transcription factor expression, in order to achieve germ layer specific gene expression patterns–a prerequisite to the differentiation of stem cells into specific cell types.

## Methods

### Ethics statement

All animal procedures and experiments were performed in accordance with Swedish animal welfare laws authorized by the Stockholm Animal Ethics Committee: Dnr N249/14.

### ChIP-seq and peak calling

As input to the ChIP-seq experiments, which were performed in duplicate according to [4], 40–60 E11.5 mouse stomachs or lungs/esophaguses were micro-dissected and fixed. SOX2 immunoprecipitation was achieved using a rabbit anti-SOX2 antibody (a kind gift from T. Edlund, Umea University, Sweden). Sequencing of Illumina Trueseq libraries of 50bp single end reads was performed on an Illumina Genome Analyzer IIx. Fastq alignment was performed to mm9 using bowtie v.0.12.7 [35], while peak calling was performed using SISSRS v.1.4 [36]. For lungs/esophaguses, the biological replicates Run00191_L1_1_130611_SN893_0191_AC2358ACXX_GGCTAC_AR011.fastq and Run00207_L3_1_131128_SN893_0207_AC2LG9ACXX_ATCACG_AR001.fastq were merged in order to create a duplicate file of approximately the same size as Run00183_L3_1_130418_SN893_0183_AD1Y1UACXX_ATCACG_AR001.fastq. Peaks were called by first assessing calling them from the merged bed files for each tissue (FDR<10^−7^ for cortex, spinal cord and stomach, and <10^−13^ for lung/esophagus, based on comparing central motif enrichment), with high background regions removed (available upon request). Then, we filtered these peaks for only those that were also called in both individual duplicate experiments in each tissue (FDR<10^−2^ for cortex, spinal cord and stomach, and 10^−4^ for lung/esophagus). The center of each peak was extended by ±100bp, and these were used for default overlap analysis in Galaxy v.16.10.rc1 [37], with all further analysis performed on these consensus peaks.

In order to confirm that our peak calling approach had revealed true SOX2 binding sites, we repeated the peak calling using MACS14 at a p-value cutoff of <10^−4^ for all experiments, and again used only peaks that overlapped in peak calls from both individual ChIP-seq repeats, as well as in the merged files. For lungs/esophaguses, Run00207_L3_1_131128_SN893_0207_AC2LG9ACXX_ATCACG_AR001.fastq and Run00183_L3_1_130418_SN893_0183_AD1Y1UACXX_ATCACG_AR001.fastq were used as duplicates.

### Animals and immunostaining

E11.5 *Sox2-Gfp* embryos (Jackson mouse stock number 017592) were photographed fresh upon dissection. BrdU treatments were performed with a single treatment of 100ug/g body weight BrdU for one hour. Antibody stains were performed using homemade rabbit anti-SOX2 (T.Edlund, Umea University, Sweden), mouse anti-Ki67 (Millipore MAB4190), rabbit anti-BrdU (Rockland 600-401-C29), and FITC conjugated goat anti-GFP (Abcam ab6662). BrdU stain denaturing was performed according to [4].

### Central enrichment, gene annotation, read coverage proportions and ChIP-seq data visualization

SOX motif central enrichment was assessed using CentriMo v.4.11.2 [38]. Nearest gene annotation was performed using GREAT v.3.0.0. The number of reads within a peak set bed file present in each ChIP-seq file was calculated using Samtools bedcov v0.1.19-96b5f2294a against merged duplicate bam files for each organ. Read proportions were calculated from bedcov results by first correcting read counts from each organ to the average reads in all the sequencing files. The number of reads calculated by bedcov for a given genomic region in a single organ was then divided by the total number of reads in all organs at that site to give a proportion of total reads in each organ. ChIP-seq data was visualized as either track alignments, averaged over 75bp, to genomic positions using IGV v.2.3.88 [39,40] or as heat maps of raw reads to bed peak files (±5kb from peak center) using Seqminer v.1.2 [41].

### Toppcluster and GO enrichment

Toppcluster [21] was used to create a Network Generator Fruchterman-Reingold output in order to visualize the top 10 GO biological process terms with between 1 and 1500 genes, which were enriched at p<0.01 with no correction in genes bound by SOX2 in the four different tissues. Gene set GO scores were assessed using Panther v11 GO biological process complete terms, with Bonferroni correction for all gene sets >550 genes. Fold change was calculated by comparing individual GO term fold enrichments to the fold enrichment of the same GO term when all genes in all groups are assessed.

### Diffbind PCAs, dendograms and heatmaps

The PCA plots were created with DiffBind. The plots are based on affinity (read count) data. A binding matrix was calculated with scores based on read counts for every sample within the binding site intervals (peaks). For the DHS PCA plots the peaks of the corresponding SOX2-ChIP were used.

### Motif enrichment and spacing analysis

Motif enrichment was performed using HOMER [42] with the findMotifsGenome.pl function (default settings), where the four remaining peak sets were used as background for the five individual HOMER runs displayed in (Fig 2A). The distances between SOX2 motifs and ZEB1, OTX1 and FOXA1 motifs was performed by searching for their core consensus sites (SOX2 = ACAAA/T, ZEB1 = CA/TCACCTG, OTX1 = TAATCCCC and FOXA1 = GTAAAC/TA) in the complete peak sets where they were identified using fastaRegexFinder.py (hosted at https://github.com/dariober/bioinformatics-cafe/tree/master/fastaRegexFinder). When the motifs were identified within the same peak, their positions were compared and assembled to find the number of occurrences for specific spacing’s. These results are presented as rolling averages of 5bp in (S4 Fig), along with the median spacing and most common spacing found in the data sets.

### RNA-seq analysis

Ten to twelve E11.5 cortices, spinal cords, stomachs and lungs/esophaguses were dissociated using a Miltenyi Biotec Neural Dissociation Kit (P; #130-092-628). 63892 cortex, 59193 spinal cord, 72151 stomach and 40074 lung/esophagus cells were then FACS sorted into triplicates on a BD Influx machine, with plots shown in S1 Fig. RNA was extracted using a Qiagen RNeasy mini kit, cDNA was made using the Smartseq2 protocol [43], and libraries were produced by following the Nextera XT manufacturer’s instructions. Sequencing of 50bp single end reads was done using an Illumina Genome Analyzer IIx. Star v2.5 [44] was used to align reads to mm9, while gene expression levels were calculated using rpkmforgenes.py [45]. Differential gene expression was assessed using Deseq2 [46], with organ specific genes showing differential expression against all other triplicate samples padj < 0.01 and fold change > 2. PCA was performed using Rstudio Prcomp.

### Gene set overlap enrichment

Overlap enrichment between different gene sets was calculated by taking the number of overlapping genes between the gene sets and dividing this by the multiple of the number of genes in the two gene sets (# overlapping genes/(# genes in set 1 x # genes in set 2)). Significance was calculated by using Rstudio prop.test between two overlapping gene sets.

### Zebrafish enhancer experiments

Common and specific SOX2 bound enhancers were selected based on their cell-type-specific binding profiles, proximity to specifically expressed genes and conservation scores, cloned into the Tol2-e1b-GFP vector and injected into one or two cell zebrafish embryos according to [7].

### Expression plasmids and luciferase assay

P19 cell luciferase assays were performed and processed as described in [8]. Luciferase reporter vectors were produced by cloning the genomic regions of *Hoxa2* +397bp (chr6:52,114,690–52,115,339), *Tm7sf2* -710bp (chr19:6,066,932–6,067,331), *Dnajc19* -115kb (chr3:34,095,332–34,095,691), *Pax3* +25kb (chr1:78,168,192–78,168,551), *Foxa2* +78kb (chr2:147,794,293–147,794,692) and *Foxp1* +11kb (chr6:99,123,301–99,123,900) into pGL3-TKMax luciferase constructs. ZEB1-flag, OTX1-flag and FOXA1-flag constructs were made by PCR amplifying the respective open reading frames from cDNA, and ligating them into pCIG expression plasmids. All other expression plasmids were reported in [4].

### *In ovo* and ex vivo stomach electroporation

*In ovo* electroporation was performed as previously described in [2], while *ex vivo* electroporation of E13.5 stomachs was performed as described with intestine in [47].

### SOX level determination

To compare SOX2 high and SOX2 low cells, each SOX2 image had the average nuclear staining intensity of at least 50 cells assayed in ImageJ [48]. SOX high cells were then defined when levels were then adjusted, such that only cells with above average nuclear staining intensity remained visible. To assign average SOX2 levels, the average nuclear SOX2 intensity of at least 50 SOX2^+^ cells was divided to the nuclear background staining of at least 50 SOX2^-^ cells.

### Co-immunoprecipitation

Co-immunoprecipitation was performed as previously described [4]. Briefly, four million HEK293 cells were seeded into a T75 flask and incubated over night. Cells were then transfected with 5μg of each expression vector using Lipofectamine 2000, according to manufacturers protocol. After over night incubation in full medium, cells were trypsinized and resuspended in 1 mL lysis buffer, frozen for 2 min at -80°C, and then incubated on ice, rocking, for 30 min. 50μL lysate was saved as input and 60μL Flag antibody bound beads (Sigma) were incubated with the lysates for 2h at 4°C. After washing, two times 30μL 3xFlag peptide was used to elute bead bound protein. To establish DNA dependency of the protein-protein interactions 100U/ml DNase I (Invitrogen) was added to the lysate for 30 min at 4°C before immunoprecipitation.

## Supporting information

S1 Fig SOX2 binding sites are shared more within germ layers than between them. (**A**) Percentage of iI67^+^ cells that are SOX2^+^ in the E11.5 cortex, spinal cord, stomach and lung/esophagus. (**B**) FACS plots of SOX2^+^ cells (encircled) sorted from dissected E11.5 SOX2-GFP cortices, spinal cords, stomachs and lung/esophagus. (**C**) Seqminer heat maps showing raw reads from all SOX2 ChIP-seqs (singlets or merged replicates) within peaks called from cortex, spinal cord, stomach or lung/esophagus SOX2 ChIP-seqs and the corresponding percentage of peaks bound in all tissues based on clustering. (**D**) The average proportion of SOX2 ChIP-seq reads derived from cortex, spinal cord, stomach and lung/esophagus experiments within the different SOX2 peak sets.
(TIF)

S2 Fig MACS14 peak calling shows a similar pattern of SOX2 binding to SISSRS. (**A**) Diffbind heat map and dendogram of the correlations between cortex, spinal cord, stomach and lung/esophagus SOX2 ChIP-seq replicates. (**B**) Central enrichment of SOX2 binding motifs in cortex, spinal cord, stomach and lung/esophagus SOX2 ChIP-seq peaks called by MACS14. The percentage of peaks that the given motifs are found centrally enriched, and their p-values are listed. (**C**) Venn diagram showing overlap between sites bound in the different SOX2 ChIP-seq experiments, as called by MACS14. 17 peaks overlapped in all four tissues. (**D**) Overlap between peaks called by MACS14 and SISSRS, with the percentage overlap with the smaller group listed.
(TIF)

S3 Fig SOX2 prebinds common, neural and endodermal genes equally in ESCs. (**A**) Seqminer heat maps showing alignment of SOX2 ChIP-seq reads from ESCs and merged replicates from cortex, spinal cord, stomach and lung/esophagus to SOX2 peaks in ESCs. The bar graph shows the percentage of ESC peaks bound in each tissue. (**B**) The percentage of common, CNS common, cortex specific, spinal cord specific, endoderm common, stomach specific and lung/esophagus specific SOX2 ChIP-seq peaks that overlap with SOX2 peaks in ESCs. (**C**) Seqminer read density-clustering heatmap of merged replicate SOX2 ChIP-seqs within peak regions called in ESCs. Stippled lines separate three clusters of ESC SOX2 peaks that are bound in all tissues (white), specifically in the CNS (red) or specifically in the endoderm (blue). (**D**) Fold enrichment and p-value scores from Panther of selected GO terms for genes within 500kb of ESC ChIP-seq peak clusters, from S3C Fig, bound in all tissues (white), specifically in the CNS (red) or specifically in the endoderm (blue).
(TIF)

S4 Fig Gene expression and SOX2 co-factors are tissue specific. (**A**) Top five HOMER *de novo* transcription factor binding motifs enriched in specific and common SOX2 ChIP-seq peak sets. Mammalian transcription factors with consensus sites matching the motif, p-values for motif enrichment and the percentage of peaks the motifs are found in are inset next to each motif. (**B**) 5bp rolling averages of distance between SOX2 motifs and those of OTX1 in cortex peaks, FOXA1 in stomach and lung/esophagus peaks and ZEB1 in all peak sets. The median distance and most common spacing are labelled on each graph. (**C**) Co-immunoprecipitation using Flag-tagged transcription factors, identified in Fig 2A as enriched in cortex specific (OTX1), lung/esophagus specific (FOXA1) or common (ZEB1) SOX2 peaks. The precipitation of Myc-tagged full-length SOX2, SOX2 C-terminus or SOX2 HMG+B-domains was analyzed. (**D**) PCA and hierarchical clustering of all RNA-seq replicates from E11.5 SOX2-GFP cortices, spinal cords, stomachs and lung/esophagus based on the most variable genes expressed above RPKM 1. (**E**) Venn diagram showing specific and overlapping gene expression based on pair-wise Deseq2 analysis padj<0.01 and fold change >2. (**F**) Fold enrichment and p-value scores from Panther of selected GO terms for genes specifically expressed in (E). (**G**) Bar graph showing the average expression of genes bound and not bound by SOX2 in the cortex, spinal cord, stomach and lung/esophagus. P-values are calculated with two sided, unpaired t-tests (* = p<0.05, ** = p<0.01, *** = p<0.001).
(TIF)

S5 Fig SOX2 bound CRMs drive expression in appropriate tissues. (**A-C**) SOX2 ChIP-seq tracks with read scale maximum values inset top left (cortex in red, spinal cord in green, stomach in blue and lung/esophagus in yellow) and reporter expression of regulatory regions commonly bound by SOX2 in both CNS and endoderm (A), specifically bound in CNS (B) or specifically bound in endoderm (C), as well as the chromosomal location of each region and statistics for the number of GFP+ fish out of total injected survivors. Arrows indicate endodermal GFP reporter expression from regions bound by SOX2 commonly and endoderm specifically.
(TIF)

S6 Fig SOX2 regulates proliferation via a conserved mechanism. (**A**) SOX2 ChIP-seq tracks of reads in cortex (red), spinal cord (green), stomach (blue) and lung/esophagus (yellow) around the *Ccnd1* promoter region with read scale maximum values inset to left. Inset is a zoom on the upstream promoter region to highlight binding to this region in the four tissues. The *Ccnd1*-409 promoter luciferase construct is repressed in a dose dependent fashion by SOX2 in P19 cells. (**B**) Percentage of electroporated cells in the 72 hpf chick spinal cord labelled by a 30 minute pulse of BrdU following epistatic manipulation of SOX2 and Wnt pathway activity. Blocking canonical Wnt-activation by B-catenin using Tcf4-dominant negative (TCF4DN) decreases proliferation, while blocking repression by TLE/GRG using Lef1-Grg binding mutant (LEF1GBM) increases proliferation regardless of SOX2 activity. (**C**) Percentage of electroporated cells in E13.5 stomach explants labelled by a 30 minute pulse of BrdU following overexpression of GFP, SOX2/LEF1GBM or dnSOXB1/TCF4DN. All error bars represent standard deviations between experiments and p-values are calculated with two sided, unpaired t-tests (* = p<0.05, ** = p<0.01, *** = p<0.001).
(TIF)

S1 Table Technical information regarding RNA-sequencing of endodermal and neural organs. (XLSX)

S2 Table Technical information regarding SOX2 ChIP-seq peaks (from SISSRS) in endodermal and neural organs. (XLSX)

S3 Table Technical information regarding SOX2 ChIP-seq peaks (from SISSRS) commonly found in endodermal and neural organs. (XLSX)

S4 Table Technical information regarding SOX2 ChIP-seq peaks (from MACS14) in endodermal and neural organs. (XLSX)

S5 Table DESeq2 specifically expressed genes in endodermal and neural organs. (XLSX)
