# Supplementary figures and images for: SOX2 regulates common and specific stem cell features in the CNS and endoderm derived organs
Source: PLoS Genet. 2018 Feb 12;14(2):e1007224. doi: 10.1371/journal.pgen.1007224 (PMC5825159; doi:10.1371/journal.pgen.1007224)

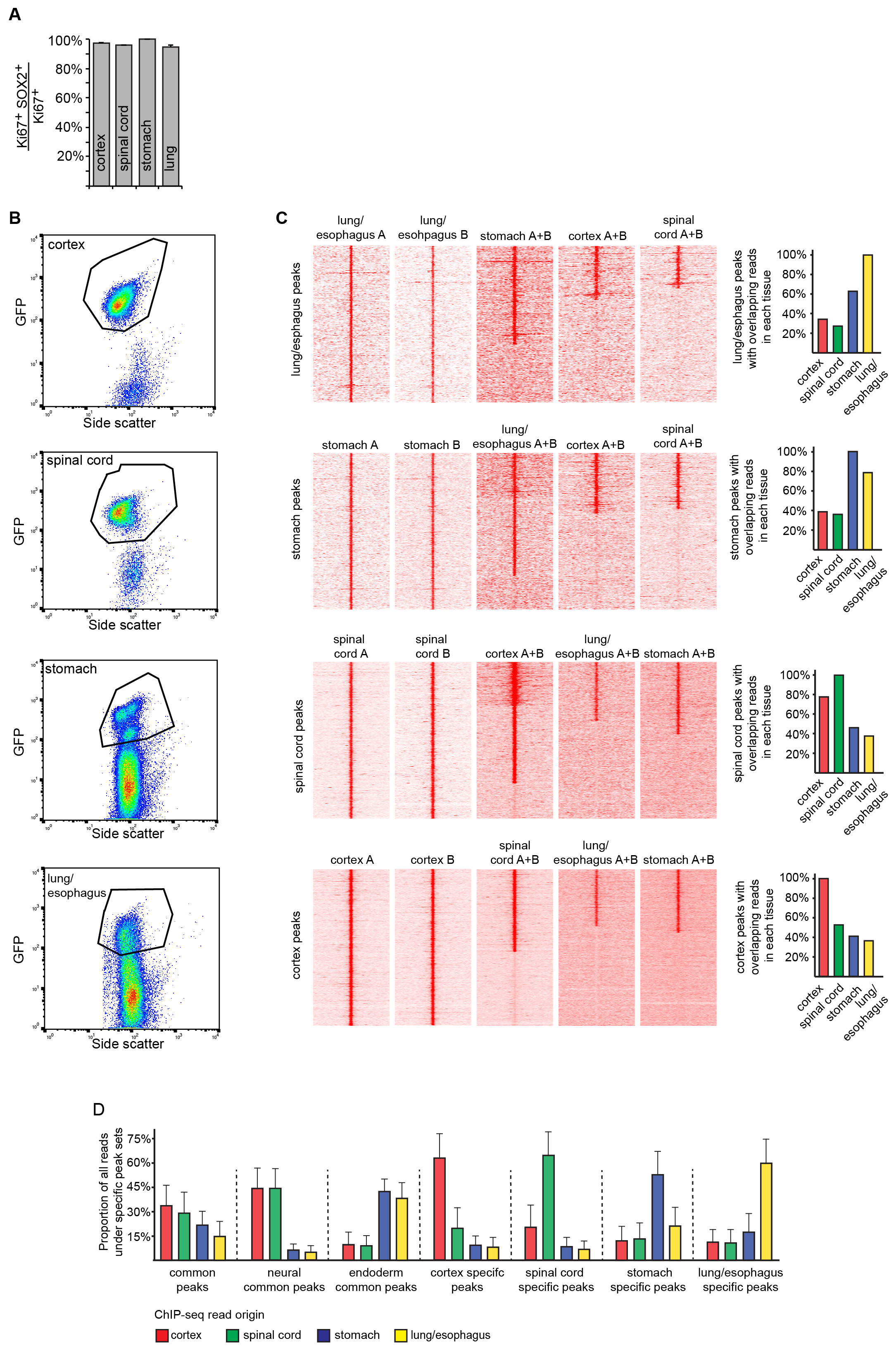

Supplement: S1 Fig — (A) Percentage of iI67+ cells that are SOX2+ in the E11.5 cortex, spinal cord, stomach and lung/esophagus. (B) FACS plots of SOX2+ cells (encircled) sorted from dissected E11.5 SOX2-GFP cortices, spinal cords, stomachs and lung/esophagus. (C) Seqminer heat maps showing raw reads from all SOX2 ChIP-seqs (singlets or merged replicates) within peaks called from cortex, spinal cord, stomach or lung/esophagus SOX2 ChIP-seqs and the corresponding percentage of peaks bound in all tissues based on clustering. (D) The average proportion of SOX2 ChIP-seq reads derived from cortex, spinal cord, stomach and lung/esophagus experiments within the different SOX2 peak sets. (TIF) [file pgen.1007224.s001.tif]

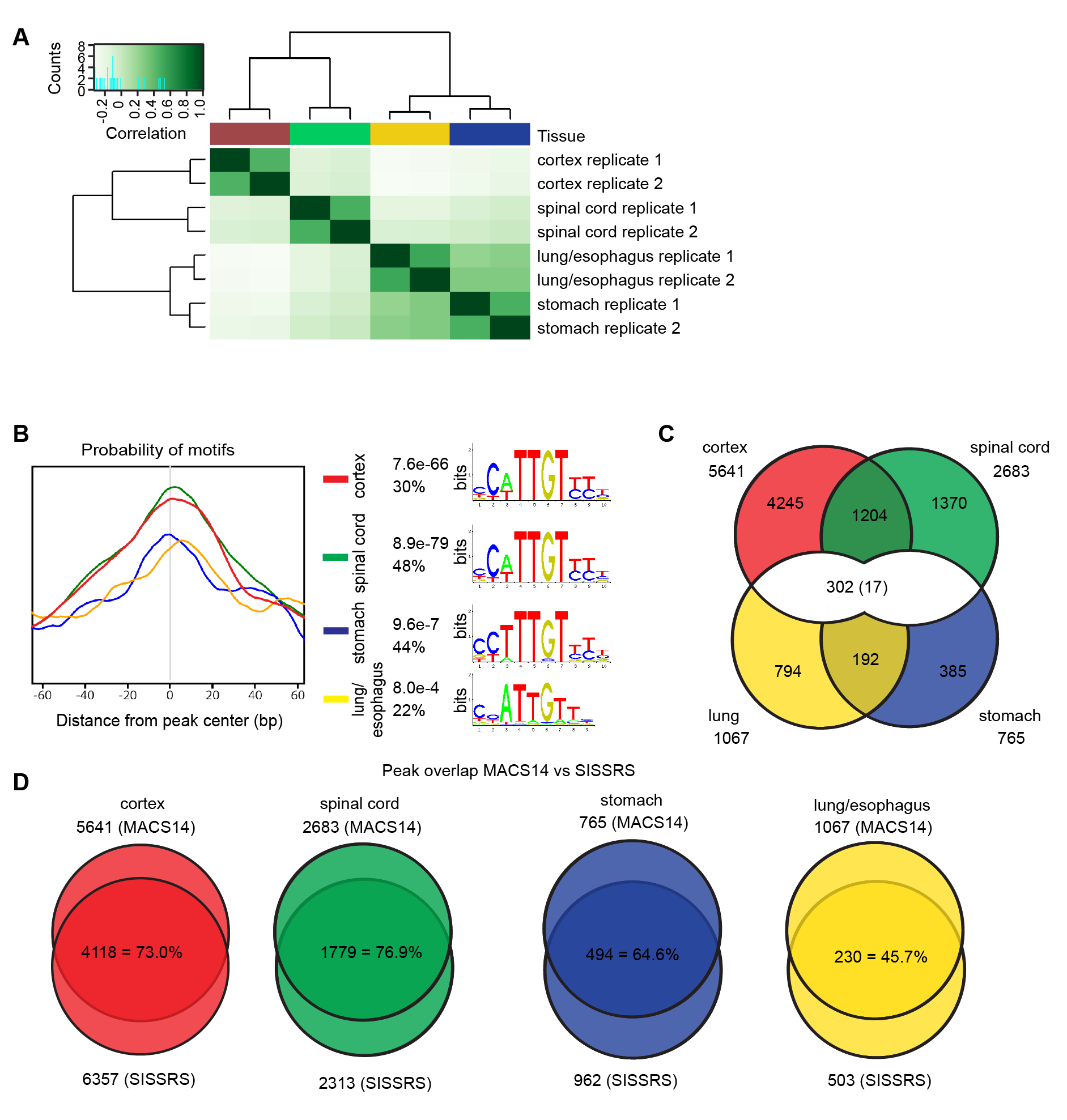

Supplement: S2 Fig — (A) Diffbind heat map and dendogram of the correlations between cortex, spinal cord, stomach and lung/esophagus SOX2 ChIP-seq replicates. (B) Central enrichment of SOX2 binding motifs in cortex, spinal cord, stomach and lung/esophagus SOX2 ChIP-seq peaks called by MACS14. The percentage of peaks that the given motifs are found centrally enriched, and their p-values are listed. (C) Venn diagram showing overlap between sites bound in the different SOX2 ChIP-seq experiments, as called by MACS14. 17 peaks overlapped in all four tissues. (D) Overlap between peaks called by MACS14 and SISSRS, with the percentage overlap with the smaller group listed. (TIF) [file pgen.1007224.s002.tif]

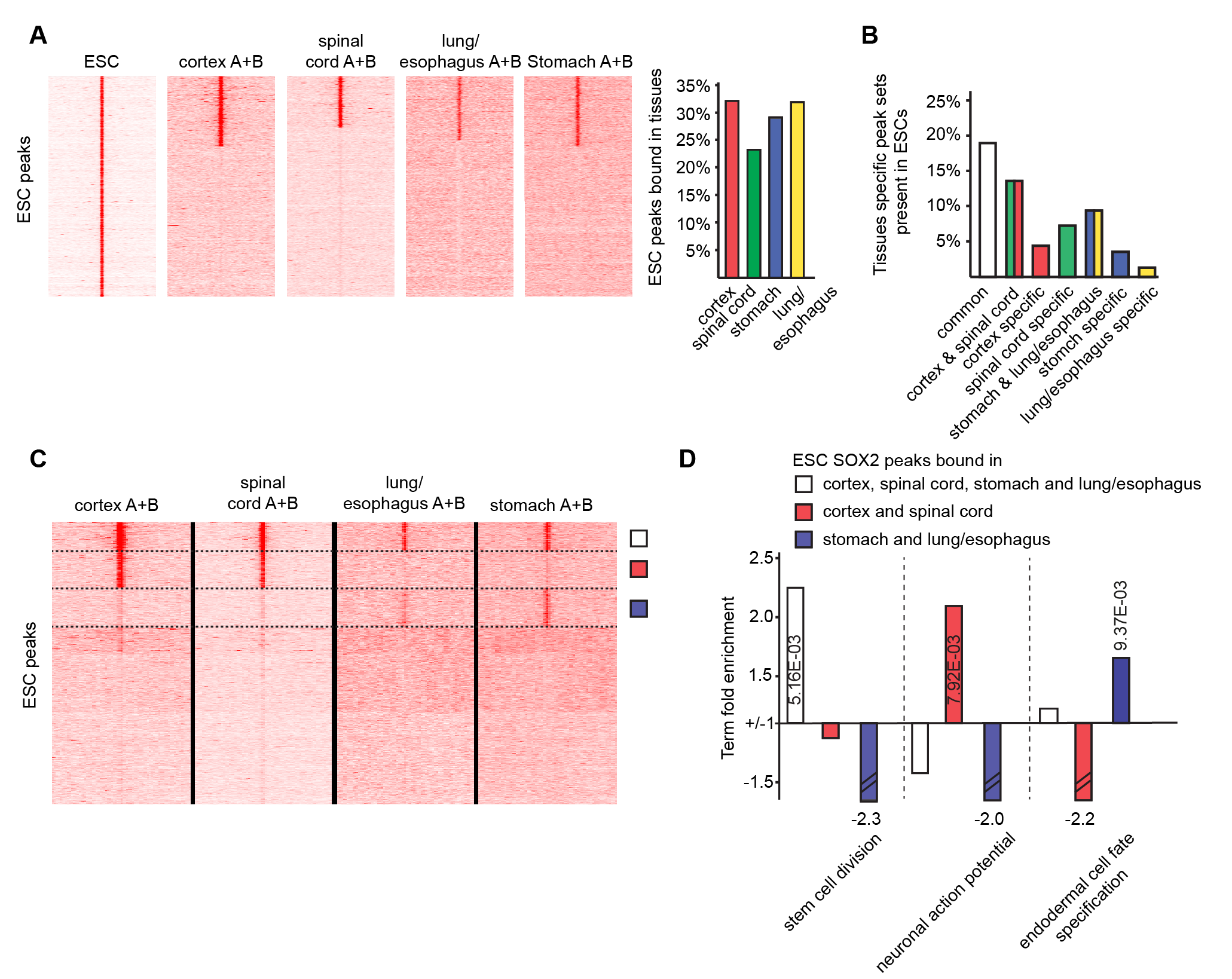

Supplement: S3 Fig — (A) Seqminer heat maps showing alignment of SOX2 ChIP-seq reads from ESCs and merged replicates from cortex, spinal cord, stomach and lung/esophagus to SOX2 peaks in ESCs. The bar graph shows the percentage of ESC peaks bound in each tissue. (B) The percentage of common, CNS common, cortex specific, spinal cord specific, endoderm common, stomach specific and lung/esophagus specific SOX2 ChIP-seq peaks that overlap with SOX2 peaks in ESCs. (C) Seqminer read density-clustering heatmap of merged replicate SOX2 ChIP-seqs within peak regions called in ESCs. Stippled lines separate three clusters of ESC SOX2 peaks that are bound in all tissues (white), specifically in the CNS (red) or specifically in the endoderm (blue). (D) Fold enrichment and p-value scores from Panther of selected GO terms for genes within 500kb of ESC ChIP-seq peak clusters, from S3C Fig, bound in all tissues (white), specifically in the CNS (red) or specifically in the endoderm (blue). (TIF) [file pgen.1007224.s003.tif]

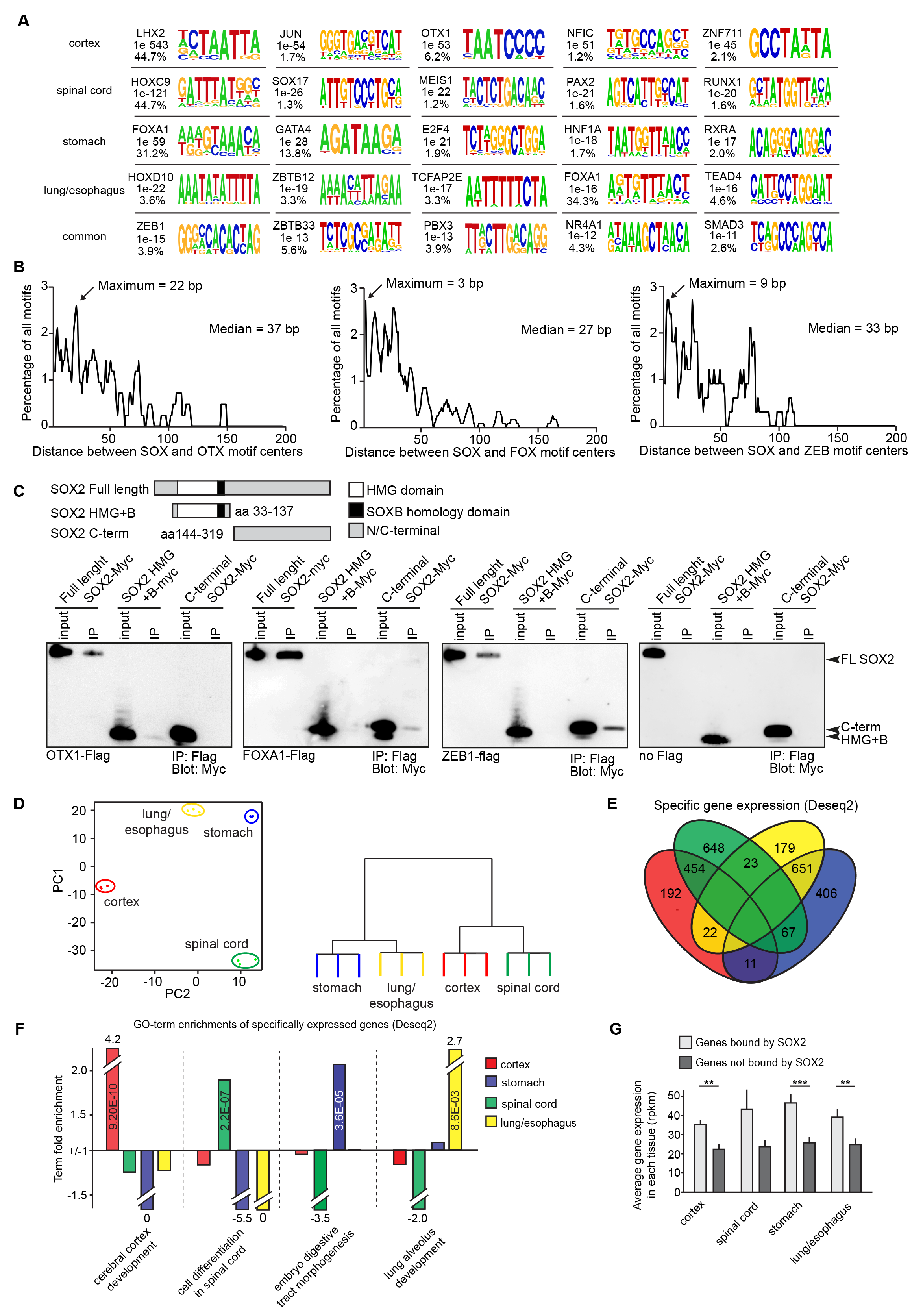

Supplement: S4 Fig — (A) Top five HOMER de novo transcription factor binding motifs enriched in specific and common SOX2 ChIP-seq peak sets. Mammalian transcription factors with consensus sites matching the motif, p-values for motif enrichment and the percentage of peaks the motifs are found in are inset next to each motif. (B) 5bp rolling averages of distance between SOX2 motifs and those of OTX1 in cortex peaks, FOXA1 in stomach and lung/esophagus peaks and ZEB1 in all peak sets. The median distance and most common spacing are labelled on each graph. (C) Co-immunoprecipitation using Flag-tagged transcription factors, identified in Fig 2A as enriched in cortex specific (OTX1), lung/esophagus specific (FOXA1) or common (ZEB1) SOX2 peaks. The precipitation of Myc-tagged full-length SOX2, SOX2 C-terminus or SOX2 HMG+B-domains was analyzed. (D) PCA and hierarchical clustering of all RNA-seq replicates from E11.5 SOX2-GFP cortices, spinal cords, stomachs and lung/esophagus based on the most variable genes expressed above RPKM 1. (E) Venn diagram showing specific and overlapping gene expression based on pair-wise Deseq2 analysis padj<0.01 and fold change >2. (F) Fold enrichment and p-value scores from Panther of selected GO terms for genes specifically expressed in (E). (G) Bar graph showing the average expression of genes bound and not bound by SOX2 in the cortex, spinal cord, stomach and lung/esophagus. P-values are calculated with two sided, unpaired t-tests (* = p<0.05, ** = p<0.01, *** = p<0.001). (TIF) [file pgen.1007224.s004.tif]

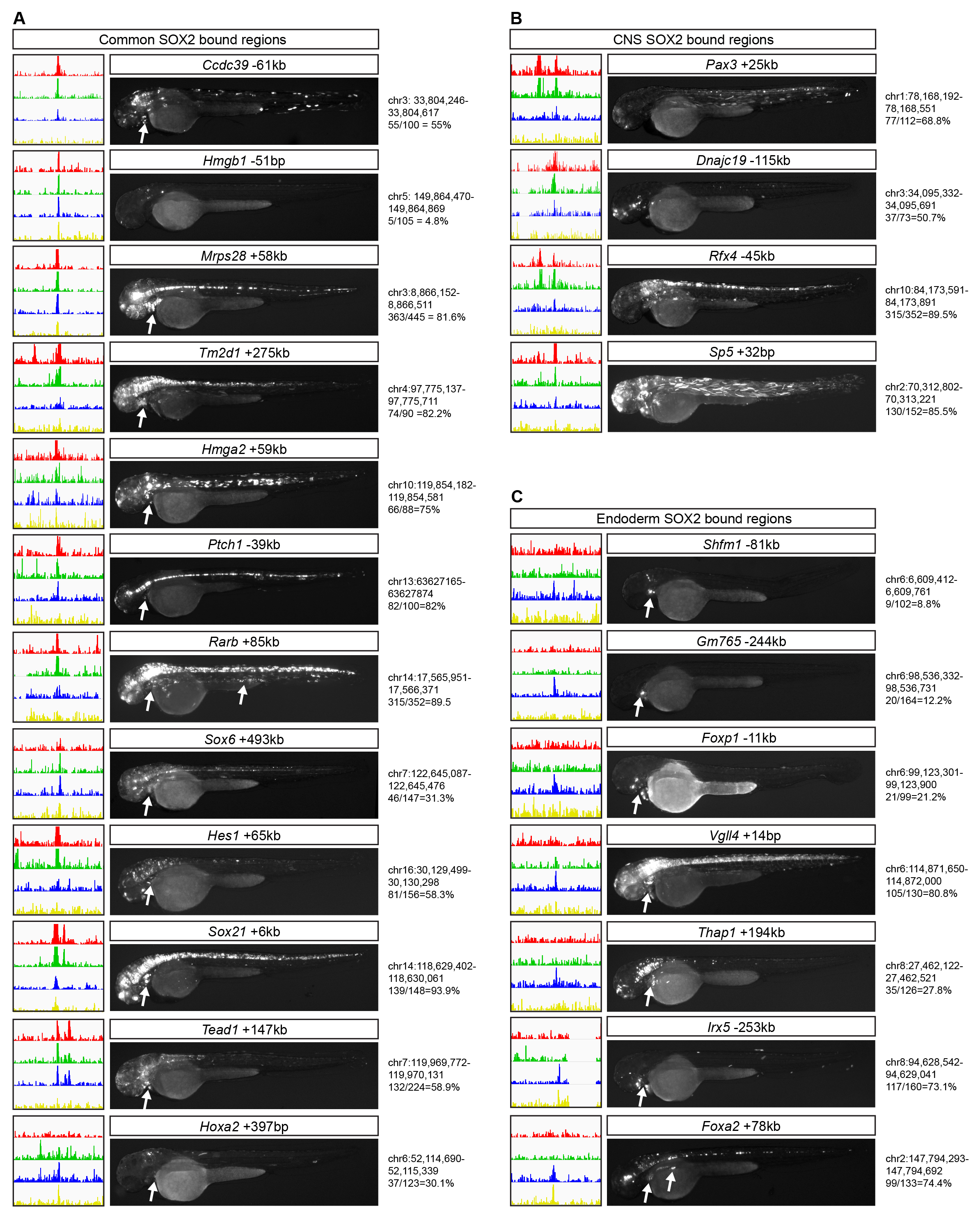

Supplement: S5 Fig — (A-C) SOX2 ChIP-seq tracks with read scale maximum values inset top left (cortex in red, spinal cord in green, stomach in blue and lung/esophagus in yellow) and reporter expression of regulatory regions commonly bound by SOX2 in both CNS and endoderm (A), specifically bound in CNS (B) or specifically bound in endoderm (C), as well as the chromosomal location of each region and statistics for the number of GFP+ fish out of total injected survivors. Arrows indicate endodermal GFP reporter expression from regions bound by SOX2 commonly and endoderm specifically. (TIF) [file pgen.1007224.s005.tif]

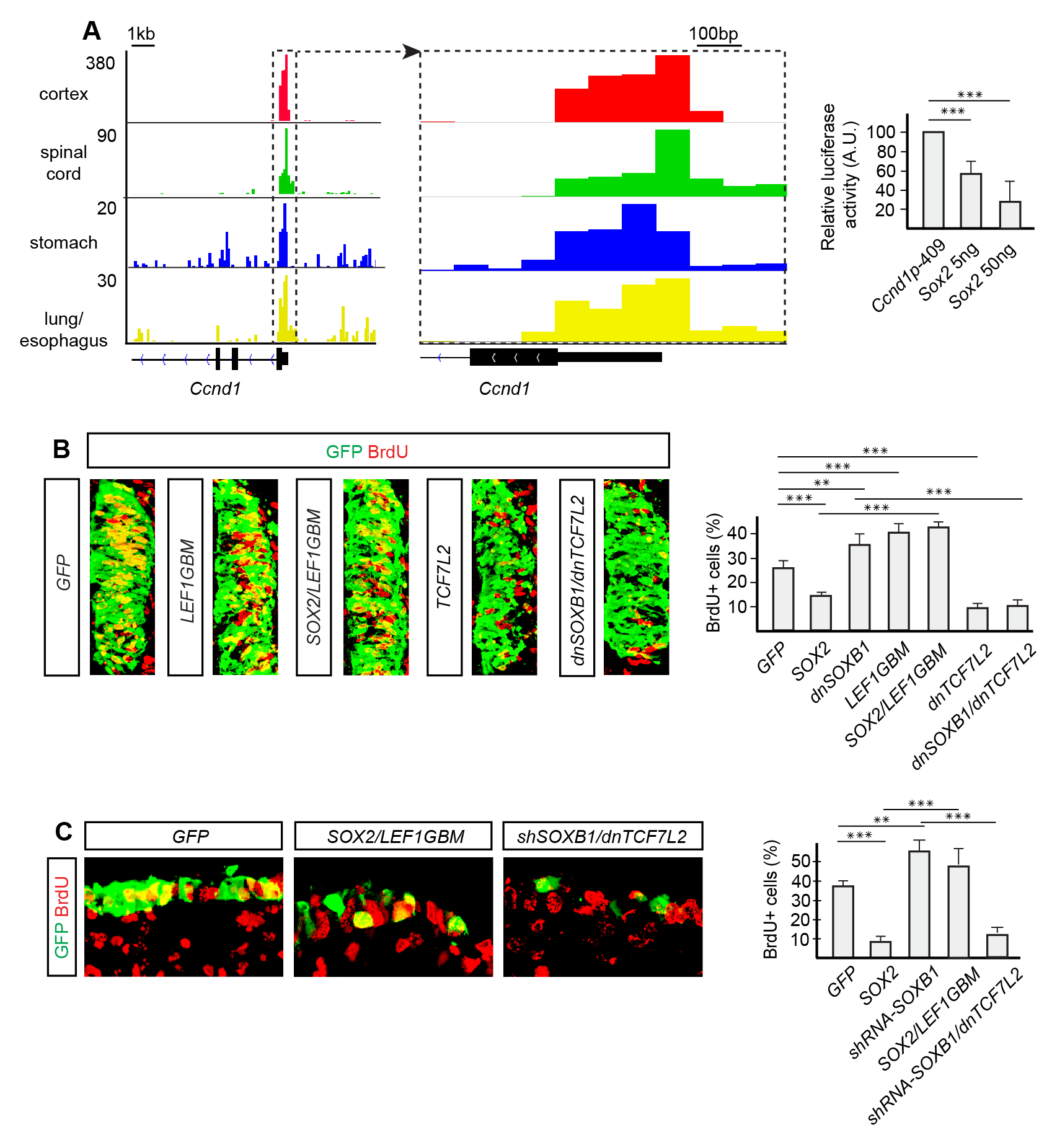

Supplement: S6 Fig — (A) SOX2 ChIP-seq tracks of reads in cortex (red), spinal cord (green), stomach (blue) and lung/esophagus (yellow) around the Ccnd1 promoter region with read scale maximum values inset to left. Inset is a zoom on the upstream promoter region to highlight binding to this region in the four tissues. The Ccnd1-409 promoter luciferase construct is repressed in a dose dependent fashion by SOX2 in P19 cells. (B) Percentage of electroporated cells in the 72 hpf chick spinal cord labelled by a 30 minute pulse of BrdU following epistatic manipulation of SOX2 and Wnt pathway activity. Blocking canonical Wnt-activation by B-catenin using Tcf4-dominant negative (TCF4DN) decreases proliferation, while blocking repression by TLE/GRG using Lef1-Grg binding mutant (LEF1GBM) increases proliferation regardless of SOX2 activity. (C) Percentage of electroporated cells in E13.5 stomach explants labelled by a 30 minute pulse of BrdU following overexpression of GFP, SOX2/LEF1GBM or dnSOXB1/TCF4DN. All error bars represent standard deviations between experiments and p-values are calculated with two sided, unpaired t-tests (* = p<0.05, ** = p<0.01, *** = p<0.001). (TIF) [file pgen.1007224.s006.tif]
